# Comparative evaluation of RT-PCR and antigen-based rapid diagnostic tests (Ag-RDTs) for SARS-CoV-2 detection: performance, variant specificity, and clinical implications

**DOI:** 10.1128/spectrum.00073-24

**Published:** 2024-04-29

**Authors:** Frank T. Aboagye, Lawrence Annison, Henry Kwadwo Hackman, Maame E. Acquah, Yvonne Ashong, Isaac Owusu-Frimpong, Bill C. Egyam, Sharon Annison, George Osei-Adjei, Samuel Antwi-Baffour

**Affiliations:** 1 Department of Medical Laboratory Technology, Faculty of Applied Sciences, Accra Technical University, Accra, Ghana; 2 Biomedical and Public Health Research Unit, Council for Scientific and Industrial Research – Water Research Institute, Accra, Ghana; 3 West African Centre for Cell Biology of Infectious Pathogens, College of Basic and Applied Sciences, University of Ghana, Accra, Ghana; 4 Department of Parasitology, Noguchi Memorial Institute of Medical Research, College of Medical Sciences, University of Ghana, Accra, Ghana; 5 Department of Molecular Biology, MDS Lancet Laboratories Ghana Limited, Accra, Ghana; 6 Department of Epidemiology and Disease Control, School of Public Health, University of Ghana, Accra, Ghana; 7 Department of Medical Laboratory Sciences, School of Biomedical and Allied Health Sciences, College of Health Sciences, University of Ghana, Accra, Ghana; London Health Sciences Centre, London, Ontario, Canada

**Keywords:** SARS-CoV-2, RNA, RT-PCR, viral load, COVID-19, RDT, antigen

## Abstract

The COVID-19 pandemic has highlighted the critical need for accurate and efficient diagnostic tools for detecting severe acute respiratory coronavirus 2 (SARS-CoV-2) infections. This study presents a comparison of two diagnostic tests: RT-PCR and antigen detection rapid diagnostic tests (Ag-RDTs). This study focused on their performance, variant specificity, and their clinical implications. A simultaneous testing of 268 samples was carried out for SARS-CoV-2 using RT-PCR and Ag-RDTs [flourescence immunoassay (FIA) and lateral flow immunoassay (LFIA)]. Viral load was quantified, and variant identification was performed using a PCR-based assay. The prevalence was found to be 30.2% using reverse transcription PCR (RT-PCR), 26.5% using FIA, and 25% using LFIA. When comparing the FIA and LFIA, the overall diagnostic performance was found to be 80.25% vs 76.54%, 96.79% vs 97.33%, 91.55% vs 90.51%, and 91.88% vs 92.56% for sensitivity, specificity, positive predictive value (PPV), and negative predictive value (NPV), respectively. Both Ag-RDTs showed a strong agreement with RT-PCR (κ = 0.78–0.80). The overall accuracies of the FIA and LFIA were 92.41% and 92.13%, respectively. The FIA showed higher sensitivity (73.68%) and PPV (92.08%) than the LFIA (65.79% and 90.56%, respectively) in asymptomatic patients. At low Ct values (<25), both Ag-RDTs had 100% sensitivity, but the sensitivity reduced to 31.82% for FIA and 27.27% for LFIA at Ct values > 30. The diagnostic sensitivity of FIA compared to LFIA for detecting the Alpha variant was 78.85% vs. 69.23% and 72.22% vs. 83.33% for the Delta variant. Both Ag-RDTs had 100% sensitivity for detecting Omicron. Both Ag-RDTs performed well in patients with high viral loads and Omicron variant infections compared to those infected with Alpha and Delta variants. This study confirms the comparable performance of RT-PCR and Ag-RDTs, specifically FIA and LFIA, for SARS-CoV-2 detection. The FIA showed higher sensitivity and PPV in asymptomatic cases, while both Ag-RDTs exhibited strong agreement with RT-PCR results. Notably, Ag-RDTs, particularly FIA, proved effective in detecting the Omicron variant and cases with high viral loads, highlighting their potential clinical utility in managing the COVID-19 pandemic.

**IMPORTANCE:**

This study is of utmost importance in providing effective responses to manage the COVID-19 pandemic. It rigorously compares the diagnostic accuracy, variant specificity, and practical considerations of reverse transcription PCR (RT-PCR) and antigen detection rapid diagnostic tests (Ag-RDTs) for severe acute respiratory coronavirus 2 (SARS-CoV-2), answering critical questions. The results of this study will help healthcare professionals choose the appropriate testing methods, allocate resources effectively, and enhance public health strategies. Given the evolution of the virus, understanding the performance of these diagnostic tools is crucial to adapting to emerging variants. Additionally, the study provides insights into logistical challenges and accessibility issues, which will contribute to refining testing workflows, particularly in resource-limited settings. Ultimately, the study’s impact extends to global healthcare, providing valuable information for policymakers, clinicians, and public health officials as they work together for mitigating the impact of the pandemic.

## INTRODUCTION

Severe acute respiratory coronavirus 2 (SARS-CoV-2), the causative agent of COVID-19, has been the cause of a global pandemic, which has challenged healthcare systems worldwide since the reporting of its first case in Wuhan, China (1). COVID-19 is a respiratory disease with symptoms similar to those of influenza, including dry cough, fever, severe headache, and tiredness (2). COVID-19 symptoms range from mild to severe respiratory diseases. Critically ill cases can result in organ dysfunction, including hepatic, renal, and cardiac injury (3). This can lead to impaired lung function, arrhythmia, and death (4). Elderly individuals, immunocompromised patients, and factors such as diabetes and hypertension have all been linked to severe illnesses and death (5, 6).

During a pandemic, the only way to control the pathogen spread is to identify affected people and isolate them as soon as possible (7). Accurate diagnosis of suspected COVID-19 through controlled testing and performance data from clinical settings is crucial in preventing the spread of the coronavirus outbreak. Unreliable tests may not detect active COVID-19 infections or can wrongly show negative results, hampering healthcare efforts.

For the identification and confirmation of COVID-19 cases, only molecular quantitative reverse transcription PCR (RT-qPCR) testing of respiratory tract samples is recommended (8). The current RT-PCR test for detecting SARS-CoV-2 is not only expensive, but it also requires skilled personnel for its execution. Additionally, the analysis process takes around 4–6 hours, resulting in a turnaround time that exceeds 24 hours. Furthermore, molecular diagnostics are not easily accessible to end-users. They are designated exclusively for skilled clinical laboratory professionals and restricted to laboratories that exhibit a medium or high level of complexity (8). Unfortunately, these resources are often in short supply in rural and remote regions of Ghana.

The COVID-19 pandemic has spread rapidly, and laboratory-based molecular testing has limited capacity. To support PCR testing, new point-of-care (POC) scaled rapid diagnostic tests have been developed. These simple diagnostic tools can diagnose COVID-19 within 30 minutes, providing immunodiagnostics that play an essential role in assessing the prevalence of the disease at the population level. Most antigen detection rapid diagnostic tests (Ag-RDTs) rely on viral nucleocapsid recognition to detect SARS-CoV-2 infection because it is the most abundant viral protein. However, the emergence of new variants of concern (VOCs) with specific nucleocapsid variations could impact the lower detection limit of these tests (9). There is a need to validate the clinical performance of rapid diagnostic tests. This study, therefore, sought to evaluate the diagnostic performance of two antigen rapid diagnostic test kits used in Ghana.

## MATERIALS AND METHODS

### Study design and sample collection

This cross-sectional study was conducted between July and December 2022 using a convenience sampling technique. Nasopharyngeal samples were taken from 286 consenting participants following the sample collection procedure described by Islam and Iqbal (10). The swabs were transported in a viral transport medium (Shanghai Focusgen Biotechnology Co., Ltd, China) to the COVID-19 Laboratory of the MDS Lancet Laboratories Ghana, East Legon, for analysis.

### Laboratory analysis

#### RNA extraction and real-time polymerase chain reaction (RT-PCR)

The Zymo Qu ick Viral RNA Extraction kit (Zymo Research Cooperation, USA) was used to extract RNA from each sample, as described by Aboagye and Acquah (11). Following nucleic acid isolation, SARS-CoV-2 RNA was amplified in a 25-µL reaction on CFX 96 1000 series Thermocycler (Bio-Rad, USA) with thermal conditions specific to the Allplex 2019 nCoV amplification kit (Seegene  Inc., Korea)  in a 0.2-mL 96-well qPCR plate, as described by Aboagye and Acquah (11). All samples with a cycle threshold (Ct) of 40 and above were considered negative for SARS-CoV-2 infection.

#### Screening for SARS-CoV-2 variants

The study screened all positive samples for the SARS-CoV-2 variants in Ghana using the Allplex SARS-CoV-2 Variant II Assay following the manufacturer’s instructions. The results are automatically analyzed using the SARS-CoV-2 Viewer V1 Trial Variant II Software (Seegene Inc., Republic of Korea) and interpreted as described by Lotti *et al*. (12).

#### Fluorescence immunoassay (FIA)

This machine-based rapid diagnostic test uses fluorescence to detect and relatively quantify the titers of the SARS-CoV-2 antigen in the nasopharyngeal specimen. The COVID-19 antigen test was performed using the STANDARD F COVID-19 FIA Ag test kit (SD Biosensor Inc., Korea) following the manufacturer’s instructions. Nasopharyngeal specimens were transferred to the buffer tube provided in the kit and sealed with a nozzle. The test kit was inserted into the fluorescence immunoassay analyzer, four drops of buffer and sample mixture were dispensed into the sample well, and the start button on the analyzer was pressed to initiate the analysis. The test kit was incubated for 15 minutes inside the analyzer (F2400), and fluorescence was measured and read as a cutoff index (COI). A COI value less than 1.00 was considered a negative result, and a COI value greater than or equal to 1.00 was considered positive for SARS-CoV-2 infection .

#### Lateral flow immunoassay (LFIA)

The LFIA for each participant was carried out using the Sure Status COVID-19 Antigen Card Test Kit (Premier Medical Corporation Ltd., India). The test kit was labeled with the participant ID on a flat surface. Ten drops (350 µL) of the buffer were added to 350 µL of the specimen in an applicator tube. The applicator tube was sealed with a nozzle and inverted ten times to homogenize the solution. Four drops of the solution were dispensed into the sample well of the test kit and incubated at room temperature for 15 minutes. The test results were read at 15 minutes and interpreted according to the manufacturer’s protocol. A test band and control band appearing together indicated a positive test for SARS-CoV-2 infection, and the presence of only the control band meant a negative result for SARS-CoV-2 infection.

### Statistical analysis

Statistical Package for the Social Sciences (SPSS) version 27 (IBM Corp., Armonk, NY, USA) and GraphPad Prism 9.0 (GraphPad Software Inc., Boston, USA) were used for analysis after data were entered into Microsoft Excel 2019 (Microsoft Corp., Washington, USA). For continuous and categorical variables, descriptive statistics were computed. For data that do not follow a normal distribution, median and interquartile range (IQR) were computed, while means with 95% CI were computed for normally distributed data. For categorical variables, proportions were also calculated. Statistical comparison between subgroups of categories was evaluated by the Mann–Whitney test, Kruskal–Wallis test distribution, and χ^2^ test where appropriate. The study classified the viral load as high (Ct <25), moderate (25 < Ct < 3 0), and low (Ct >30), as described elsewhere (13). Sensitivity, specificity, positive predictive value (PPV), and negative predictive value (NPV) were computed using MedCalc Statistical Software version 22.001 (MedCalc Software Ltd, Ostend, Belgium) according to different SARS-CoV-2 prevalence rates. The Cohen’s kappa (κ) value was used to assess the comparability of the overall diagnostic performance of the Ag RDT kits: poor for values between 0.0 and 0.20, fair for values between 0.21 and 0.40, moderate for values between 0.41 and 0.60, strong for values between 0.61 and 0.80, and nearly perfect for values between 0.81 and 1.00 (14). More so, the area under curve (AUC) for the receiver operator curve (ROC) analysis was classified as unsatisfactory if AUC <0.7, acceptable if 0.7 ≤ AUC < .8, excellent if 0.8 ≤ AUC < .9, and outstanding if AUC ≥0.9 (15).

## RESULTS

### Study population characteristics and disease prevalence

**Fig 1:**
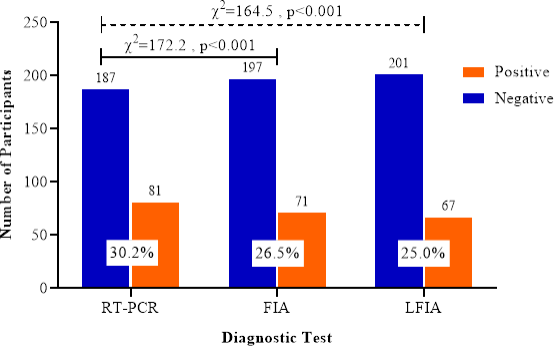
Prevalence of SARS-CoV-2 infection determined with RT-PCR and Ag-RDTs.

The majority of the 268 participants were men (51.5%, 213/268), and the overall median age was 39 years (IQR: 25.0–53.8). Most participants were asymptomatic (79.5%, 213/268). Of the 268 participants, RT-PCR detected 81 (30.2%, CI_95_: 24.0–37.6) cases of SARS-CoV-2 infection. Regarding the Ag-RDTs, 71 (26.5%, CI_95_: 20.7–33.4), and 67 (25.0%, CI_95_: 19.4–31.8) cases of SARS-CoV-2 infections were diagnosed using the FIA and LFIA, respectively, as shown in Fig. 1. There was a statistically significant difference (p < 0.001) in the reported prevalence of SARS-CoV-2 between RT-PCR and the antigen tests.

### Diagnostic performance of Ag-RDTs

Out of the 81 RT-PCR-positive cases, the FIA test correctly classified 65 participants as positive for SARS-CoV-2 infection. The LFIA also correctly classified 62 samples as positive. Considering RT-PCR as the index test, the FIA reported 6 false positives and 16 false negatives. Similarly, 5 false positives and 19 false negatives were obtained using the LFIA (Table 1).

**TABLE 1:** Overall RT-PCR and Ag-RDT results in contingency table

Antigen tests		RT-PCR		Total antigen results
		Positive	Negative	
FIA (SD-Biosensor)	Positive	65	6	71
	Negative	16	181	197
Total RT-PCR results		81	187	268
LFIA (Sure Status)	Positive	62	5	67
	Negative	19	182	201
Total RT-PCR results		81	187	268

The diagnostic performance of the FIA and LFIA is described in detail in Table 2. The sensitivity and specificity of the FIA test kit were 80.25% (CI_95_: 70.30–87.46) and 96.79% (CI_95_: 9.18–98.52), respectively. The LFIA shows a sensitivity of 76.54% (CI_95_: 65.82–85.25) and a specificity of 97.33% (CI_95_: 93.87–99.13). The PPV recorded in this study is 91.55% (CI_95_: 82.76–94.94) and 90.51% (CI_95_: 779.94–95.81) for FIA and LFIA, respectively (Table 2). The FIA shows a 91.88% (CI_95_: 87.22–94.94) NPV, while the LFIA shows an NPV of 92.56% (CI_95_: 89.35–94.56). The Kappa (κ) coefficient measure of agreement between RT-PCR and Ag-RDTs was 0.80 (CI_95_: 0.72–0.88, *P* < 0.001) for the FIA and 0.78 (CI_95_: 0.69–0.86, *P* < 0.001) for the LFIA.

**TABLE 2:** Overall diagnostic performance of Ag-RDTs using RT-PCR as the gold standard^*a*^

	FIA	LFIA
Sensitivity	80.25 (70.30–87.46)	76.54 (65.82–85.25)
Specificity	96.79 (93.18–98.52)	97.33 (93.87–99.13)
LR+	25.01 (11.30–55.36)	28.63 (11.96–68.54)
LR–	0.20 (0.13–0.32)	0.24 (0.16–0.36)
PPV	91.55 (82.76–96.07)	90.51 (79.94–95.81)
NPV	91.88 (87.22–94.94)	92.56 (89.35–94.86)
Accuracy	92.41 (88.57–95.28)	92.13 (88.23–95.06)
Cohen’s kappa (κ) standard error (*P*-value)	0.80 (0.72–0.88) 0.041 (<0.001)	0.78 (0.69–0.86) 0.043 (<0.001)

^*a*^ LR+/–: positive and negative likelihood ratio; PPV: positive predictive value, NPV: negative predictive value; AUC= area under the curve; FIA = SD Biosensor Standard F COVID-19 Ag Test; LFIA = Sure Status COVID-19 Antigen Card Test.

**Fig 2:**
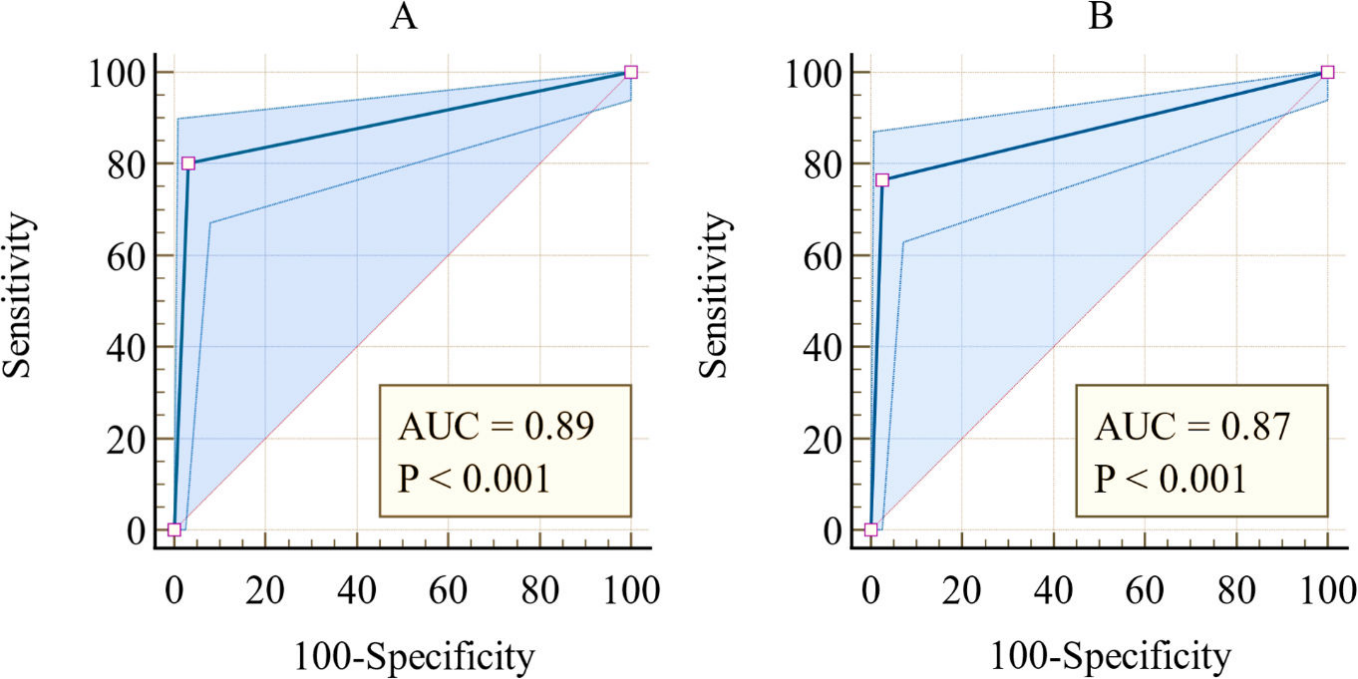
ROC analysis to evaluate the diagnostic value of the (**A**) FIA and (**B**) LFIA for SARS-CoV-2 detection.

As shown in Fig. 2, ROC curve analysis was performed to determine the AUC of the antigen level, allowing the distinction of the SARS-CoV-2 infection status. The AUC for FIA was 0.89 (CI_95_:0.83–0.94, *P* < 0.001), and that of LFIA was 0.87 (CI_95_: 0.82–0.91, *P* < 0.001). Comparatively, no significant difference was observed between the AUCs of FIA and LFIA (*P* = 0.397).

### SARS-CoV-2 viral load and Ag-RDT diagnostic performance

**Fig 3:**
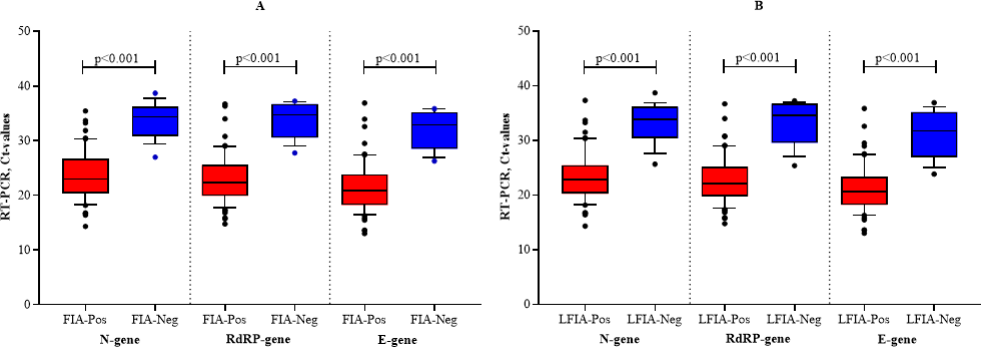
Ct values for SARS-CoV-2 N-gene, RdRP gene, and E-gene in FIA- (**A**) and LFIA-(**B**) positive and negative cases. The horizontal central line inside the box represents the median. The boxes represent the interquartile range (lower, 25th, and upper, 75th percentile). The lower and upper whiskers represent minimum and maximum Ct-values, respectively. FIA = SD Biosensor Standard F COVID-19 Ag Test; LFIA = Sure Status COVID-19 Antigen Card Test.

The study tested the diagnostic performance of the Ag-RDTs concerning the severity of the disease. To understand the analytical performance, we analyzed the results by correlating RT-PCR Ct-values with the outcomes of the Ag-RDT results (Fig. 3). The RT-PCR targets three genes: the nucleocapsid gene (N-gene), envelope gene (E-gene), and the RNA-dependent RNA polymerase (RdRP). The median N-gene Ct-value for FIA-positive cases was 23.00 (IQR: 20.34–26.74), and for FIA-negative cases, the median N-gene Ct-value was 34.33 (IQR: 30.83–38.71). RdRP gene median values in FIA-positive and FIA-negative cases were 22.31 (IQR: 19.93–25.54) and 34.70 (IQR: 30.59–36.70), respectively. The median E-gene Ct-value for FIA-positive cases was 20.91 (IQR: 18.26–23.83) and 32.92 (IQR: 28.52–35.19) for FIA-negative cases (Fig. 3). The N-gene, RdRP-gene, and E-gene median Ct-values for LFIA-positive cases were 22.79 (IQR: 20.25–25.48), 22.09 (IQR: 19.78–25.17), and 20.64 (IQR: 18.22–23.31), respectively. In LFIA-negative cases, the N-gene median Ct-value was 33.78 (IQR: 30.38–36.15) and the RdRP-gene and E-gene median Ct-values were 34.57 (IQR: 29.56–36.70) and 31.71 (IQR: 26.91–35.71), respectively (Fig. 3). A statistically significant difference (*P* < 0.001) was observed between the RT-PCR^+^/Ag-RDTs^+^ and RT-PCR^+^/Ag-RDTs^–^.

**Fig 4:**
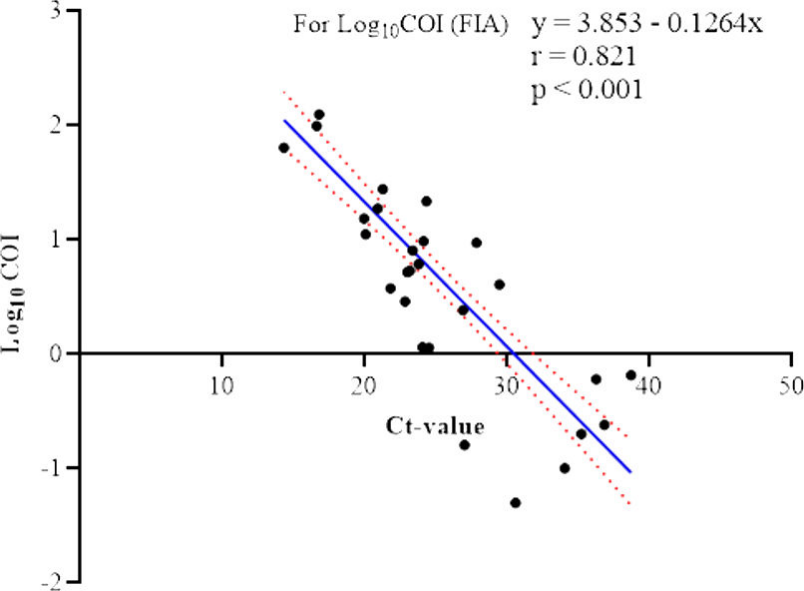
Relationship between the COI of the FIA and Ct-values of RT-PCR of PCR-positive nasopharyngeal specimens. The diagonal blue line is the linear regression fit to Ct values for log_10_-transformed antigen COIs. The red dots above and below the blue line indicate the mean error.

The COI range for the positive RT-PCR samples was 0.02–130.7, with a median value of 5.32 (IQR: 1.23–23.38). Figure 4 shows an inverse relationship between the RT-PCR Ct-values (a proxy for viral load) and the COI of the FIA (which represents the titer of the antigen detected). The relationship between Ct-value and COI in this study was statistically significant (*P* < 0.001).

We further analyzed the diagnostic performance of the Ag-RDTs against viral load using Ct-values as a proxy for viral load. As shown in Table 3, the FIA sensitivity in clinical samples with Ct-values < 25, 25–30, and >30 was 100.00% (CI_95_: 92.29–100.00), 92.31% (CI_95_: 66.69–99.61), and 31.82% (CI_95_: 13.87–54.87), respectively. The specificity for the FIA in samples with a Ct-value <25 was 88.74% (CI_95_: 83.90–92.25). At Ct-values between 25 and 30 and Ct-values > 30, the FIA shows a specificity of 76.86% (CI_95_: 71.31–81.62) and 73.98% (CI_95_: 68.03–79.35), respectively. The PPV and NPV for FIA were high in samples with a Ct-value <25. Table 3 summarizes the diagnostic performance of the FIA with respect to viral load (Ct-values).

**TABLE 3:** Performance of the FIA across groups of RT-PCR Ct-values^*a*^

	Ct-value		
	< 25	25–30	> 30
Sensitivity	100.00 (92.29–100.00)	92.31 (66.69–99.61)	31.82 (13.87–54.87)
Specificity	88.74 (83.90–92.25)	76.86 (71.31–81.62)	73.98 (68.03–79.35)
LR+	8.88 (6.14–12.85)	3.99 (3.04–5.24)	1.22 (0.64–2.34)
LR–	0.00	0.10 (0.02–0.66)	0.92 (0.69–1.24)
PPV	77.11 (69.96–82.97)	16.90 (9.94–27.26)	31.69 (19.54–46.98)
NPV	100 (98.09–100.00)	99.49 (97.18–99.97)	74.15 (68.74–78.91)
Accuracy	91.72 (87.76–94.73)	80.96 (75.74–85.48)	62.81 (56.72–68.61)
AUC	0.94 (0.91–0.97)	0.85 (0.80–0.89)	0.53 (0.47–0.59)

^*a*^ LR+/–: positive and negative likelihood ratio; PPV: positive predictive value; NPV: negative predictive value; AUC= area under the curve; FIA = SD Biosensor Standard F COVID-19 Ag Test.

Table 4 gives a detailed description of the diagnostic performance of the LFIA across the Ct-value groups. For the LFIA, the sensitivity in samples with Ct <25 was 100.00% (CI_95_: 92.29–100.00). For samples with Ct-values 25–30 and >30, the LFIA shows a sensitivity of 76.92% (CI_95_: 46.19–94.96) and 27.27% (CI_95_: 10.27–50.22), respectively. Similarly, the LFIA shows a decline in specificity with decrease in viral load decreases (Table 4). PPV [77.90% (CI_95_: 70.11–84.11)] and NPV [100.00% (CI_95_: 98.12–100.00)] are high in samples with Ct-values <25.

**TABLE 4:** Performance of the LFIA across groups of RT-PCR Ct-values^*a*^

	Ct-value		
	< 25	25–30	> 30
Sensitivity	100.00 (92.29–100.00)	76.92 (46.19–94.96)	27.27 (10.27–50.22)
Specificity	90.54 (85.90–94.05)	77.65 (72.03–82.61)	75.20 (69.32–80.47)
LR+	10.57 (7.04–15.88)	3.44 (2.36–5.01)	1.10 (0.54–2.25)
LR–	0	0.30 (0.11–0.80)	0.97 (0.74–1.26)
PPV	77.90 (70.11–84.11)	53.43 (44.07–62.54)	28.40 (16.23–44.80)
NPV	100 (98.12–100.00)	90.99 (78.87–96.47)	74.15 (68.74–78.91)
Accuracy	92.91 (89.14–95.67)	77.46 (71.98–82.32)	62.50 (56.41–68.32)
AUC	0.95 (0.92–0.98)	0.77 (0.72–0.82)	0.51 (0.45–0.57)

^*a*^ LR+/–: positive and negative likelihood ratio; PPV: positive predictive value; NPV: negative predictive value; AUC= area under the curve; LFIA = Sure Status COVID-19 Antigen Card Test.

### Clinical status and Ag-RDT performance

The study further analyzed the influence of participants’ clinical status (symptomatic or asymptomatic) on the diagnostic capacity of the Ag-RDTs. Table 5 gives a detailed description of the diagnostic performance of Ag-RDTs in detecting SARS-CoV-2 infection in symptomatic and asymptomatic participants. In symptomatic participants, the FIA and LFIA show the same sensitivity of 86.05% (CI_95_: 72.07–94.70). However, the sensitivity of the FIA (73.68%, CI_95_: 56.90–86.60) varied from that of LFIA (65.79%, CI_95_: 48.64–80.37) in asymptomatic participants (Table 5). The specificity of the FIA in detecting SARS-CoV-2 infection in symptomatic participants was 83.33% (CI_95_: 51.58–97.91), and that of the LFIA was 91.67% (CI_95_: 61.52–99.79). The PPV and NPV of the FIA in symptomatic participants were 65.05% (CI_95_: 34.31–86.90) and 94.31% (CI_95_: 88.32–97.32), respectively. The LFIA shows a PPV and an NPV of 77.49% (CI_95_: 34.43–95.76) and 95.17% (CI_95_: 90.20–97.69), respectively, in symptomatic participants, as shown in Table 5.

**TABLE 5:** Performance of Ag-RDTs in diagnosing SARS-CoV-2 in symptomatic and asymptomatic participants^*a*^

	FlA		LFIA	
	Symptomatic	Asymptomatic	Symptomatic	Asymptomatic
Sensitivity	86.05 (72.07–94.70)	73.68 (56.90–86.60)	86.05 (72.07–94.70)	65.79 (48.64–80.37)
Specificity	83.33 (51.58–97.91)	97.71 (94.25–99.37)	91.67 (61.52–99.79)	97.71 (94.25–99.37)
LR+	5.16 (1.45–18.40)	32.24 (12.01–86.51)	10.33 (1.58–67.69)	28.78 (10.64–77.89)
LR–	0.17 (0.08–0.37)	0.27 (0.16–0.46)	0.15 (0.07–0.33)	0.35 (0.23–0.54)
PPV	65.05 (34.31–86.90)	92.08 (81.24–96.89)	77.49 (34.43–95.76)	90.56 (78.00–96.29)
NPV	94.31 (88.32–97.32)	91.15 (85.81–94.61)	95.17 (90.20–97.69)	89.55 (84.64–93.02)
Accuracy	84.05 (71.68–92.53)	91.35 (86.73–94.76)	90.26 (79.22–96.60)	89.73 (84.85–93.46)
AUC	0.85 (71.68–92.53)	0.86 (0.80–0.90)	0.89 (0.78–0.96)	0.82 (0.76–0.87)

^*a*^ LR+/–: positive and negative likelihood ratio; PPV: positive predictive value; NPV: negative predictive value; AUC= area under the curve; FIA = SD Biosensor Standard F COVID-19 Ag Test; LFIA = Sure Status COVID-19 Antigen Card Test.

### SARS-CoV-2 variants and Ag-RDT performance

The study further sought to determine the impact of SARS-CoV-2 variants (Alpha, Delta, and Omicron) on the diagnostic capacity of the Ag-RDTs. As shown in Table 6, the sensitivity of the FIA in detecting Alpha variants in participants was 78.85% (CI_95_: 65.30–88.94), and in detecting the Delta and Omicron variants, the sensitivity of FIA was 72.22% (CI_95_: 46.52–90.31) and 100.00% (CI_95_:71.51–100.00), respectively. In addition, the PPV and NPV of the FIA in detecting infections associated with the Alpha variant were 29.19% (CI_95_: 24.90–33.88) and 65.32% (CI_95_: 42.04–83.03), respectively. In infections associated with the Delta and Omicron variants, the PPV of the FIA was 27.46% (CI_95_: 21.76–34.00) and 35.93% (CI_95_: 33.05–38.92), respectively, and NPV values of 59.23% (CI_95_: 36.71–78.44) and 100.00% (CI_95_: 79.41–100.00) also, respectively Table 6.

**TABLE 6:** Diagnostic performance of the FIA under variant-specific SARS-CoV-2 infection^*a*^

	SARS-CoV-2 variants		
	Alpha	Delta	Omicron
Sensitivity	78.85 (65.30–88.94)	72.22 (46.52–90.31)	100.00 (71.51–100.00)
Specificity	17.24 (5.85–35.76)	17.46 (9.05–29.10)	22.86 (13.67–34.45)
LR+	0.95 (0.77–1.19)	0.88 (0.64–1.19)	1.27 (1.14–1.47)
LR–	1.23 (0.47–3.19)	1.59 (0.64–3.99)	0.00
PPV	29.19 (24.90–33.88)	27.46 (21.76–34.00)	35.93 (33.05–38.92)
NPV	65.32 (42.04–83.03)	59.23 (36.71–78.44)	100.00 (79.41–100.00)
Accuracy	35.85 (25.49–47.27)	34.00 (23.84–45.37)	46.15 (35.01–57.59)
AUC	0.48 (0.37–0.59)	0.45 (0.34–0.56)	0.61 (0.50–0.72)

^*a*^ LR+/–: positive and negative likelihood ratio; PPV: positive predictive value; NPV: negative predictive value; AUC= area under the curve; FIA = SD Biosensor Standard F COVID-19 Ag Test.

Under the conditions of variant-specific SARS-CoV-2 infections, the sensitivity of the LFIA in detecting infection associated with Alpha, Delta, and Omicron variants was 69.23% (CI_95_: 54.90–81.28), 83.33% (CI_95_: 58.58–96.42) and 100.00% (CI_95_: 71.51–100.00), respectively. The LFIA was 10.35% (CI_95_: 2.19–27.35) specific in detecting SARS-CoV-2 infection associated with the Alpha variant; for the detection of Delta and Omicron variants, the specificity of the LFIA was 25.40% (CI_95_: 15.27–37.94) and 27.14% (CI_95_: 17.20–39.10), respectively. Table 7 gives a detailed description of the diagnostic capacity of the LFIA under variant-specific SARS-CoV-2 infections.

**TABLE 7:** Diagnostic performance of the LFIA under variant-specific SARS-CoV-2 infection^*a*^

	SARS-CoV-2 variants		
	Alpha	Delta	Omicron
Sensitivity	69.23 (54.90–81.28)	83.33 (58.58–96.42)	100.00 (71.51–100.00)
Specificity	10.35 (2.19–27.35)	25.40 (15.27–37.94)	27.14 (17.20–39.10)
LR+	0.77 (0.62–0.96)	1.12 (0.87–1.44)	1.37 (1.19–1.58)
LR–	2.97 (0.95–9.36)	0.66 (0.22–2.00)	0
PPV	25.04 (21.15–29.38)	32.58 (27.31–38.34)	37.26 (33.98–40.66)
NPV	43.73 (19.80–70.98)	77.89 (53.56–91.49)	100.00 (82.35–100.00)
Accuracy	28.13 (18.70–39.22)	42.89 (31.95–54.38)	49.15 (37.86–60.50)
AUC	0.40 (0.29–0.51)	0.54 (0.43–0.66)	0.64 (0.52–0.74)

^*a*^ LR+/–: positive and negative likelihood ratio; PPV: positive predictive value; NPV: negative predictive value; AUC= area under the curve; LFIA = Sure Status COVID-19 Antigen Card Test.

## DISCUSSION

In the wake of the global spread of COVID-19, the “test–trace–isolate” mantra remains the best strategy for controlling the spread of the virus and possibly its complete eradication. In light of this, reliable diagnostic tools are required in the testing. Ag-RDTs are the plausible options as they are less expensive, available, and less laborious compared to RT-PCR. This study evaluated two Ag-RDTs: the STANDARD F COVID-19 Test (FIA, from SD Biosensor Inc., Korea) and the Sure Status COVID-19 Card Test (LFIA, from Premier Medical Corporation Ltd., India). The prevalence of SARS-CoV-2 infection reported using the FIA was higher than that of the LFIA. Nonetheless, both Ag-RDTs differed significantly from RT-PCR regarding the reported prevalence (Fig. 1). In Uganda, the reported prevalence of SARS-CoV-2 using the LFIA was 15.7% (16), lower than the 25% reported in this study; however, similar to this study, the prevalence reported using RT-PCR (36.1%) was higher than that using the LFIA (15.77%).

### Overall diagnostic performance of Ag-RDTs

The Ag-RDTs showed varying performance levels in this evaluation relative to the reference method. The FIA and LFIA present a nearly perfect agreement (κ) with RT-PCR (Table 2). Nonetheless, the FIA (sensitivity: 80.25% and specificity: 96.79%) shows a higher sensitivity than the LFIA (sensitivity: 76.54% and specificity: 97.33%) but a lower specificity in detecting SARS-CoV-2 infections. In its preliminary guidelines on using SARS-CoV-2 rapid antigen kits, the WHO recommends using kits with a sensitivity of ≥80% and specificity of ≥97% (8). The findings of this study show that the FIA demonstrates better clinical performance, consistent with results reported by similar independent evaluations (17–20).

Participants who tested positive using the Ag-RDTs had a significant likelihood of having COVID-19, with the PPV of both the LFIA and FIA above 90% in a 25% and 26% prevalence setting, respectively (Table 2). Furthermore, there was a generally strong agreement between the Ag-RDTs and RT-PCR, considering the kappa values reported (Table 2). Furthermore, this study reported an excellent discriminatory ability of the Ag-RDTs (Fig. 2). The FIA and LFIA have AUC values above 0.80, indicating excellent ability to distinguish between positive and negative COVID-19 cases using Ag-RDTs.

### Disease severity and Ag-RDT diagnostic performance

We present a significant and linear inverse relationship between COI and Ct-values in this study (Fig. 4). This denotes that when the Ct-value decreases, the outcome of the FIA test, measured as the COI, will increase. To our knowledge, this is the first report showing a linear correlation between viral loads in nasopharyngeal specimens (proxied by Ct-values) and the relative quantity of viral antigen detection (measured as COI values) in Ghana. Other studies have reported a statistically significant correlation between the COI and Ct-value (21, 22).

The study further shows a significant difference in Ct-values between positive and negative samples tested using Ag-RDTs, considering viral load influence (Fig. 3), which is consistent with previous studies (9, 23). This study indicates that both Ag-RDTs showed 100% sensitivity under high viral load conditions (Ct <25) but a decrease in sensitivity with a decrease in viral load (Tables 3 and 4). In accordance with this study, several independent evaluations have indicated similar results (20, 24–27) and further support the manufacturer’s assertion. On the contrary, other studies have reported lower sensitivity for LFIA samples of Ct <25 (28–30). Similar to the pattern observed for sensitivity, the specificity, PPV, NPV, and accuracy of the Ag-RDTs decline as the viral load decreases.

### Impact of clinical status on Ag-RDT diagnostic performance

The containment of COVID-19 became a challenge because of the presence of asymptomatic infected persons globally. The ability of a diagnostic test to accurately diagnose SARS-CoV-2 infection in asymptomatic patients is crucial as this category of patients is known to be the source of infection spread in Ghana. In this study, the FIA showed better sensitivity with a higher PPV in asymptomatic participants than the LFIA (Table 5). The diagnostic performance of Ag-RDTs was affected by symptomatic status. Several studies have shown that Ag-RDTs have lower sensitivity in asymptomatic individuals than in symptomatic patients. For example, a study by Kiyasu *et al.* (31) found that the sensitivity of the QuickNavi-COVID19 Ag test was significantly lower for asymptomatic individuals than for symptomatic patients. Similarly, other studies have reported that false-negative results were detected in asymptomatic individuals with Ag-RDTs and later underwent PCR testing (32, 33). When used for universal screening of asymptomatic individuals, the FIA Ag-RDT has a high diagnostic yield with limited false-positives (34). Therefore, the combination of silver amplification technology and specific monoclonal antibodies against SARS-CoV-2 NP contributed to the better performance of FIA Ag-RDT in terms of sensitivity and PPV for SARS-CoV-2 diagnosis in asymptomatic patients.

### Ag-RDT diagnostic capacity for SARS-CoV-2 variants

Most Ag-RDT validation studies were conducted before the emergence of different concern variants. Both Ag-RDTs showed a higher sensitivity for detecting Omicron than for the Alpha and Delta variants. However, the FIA had a better sensitivity for detecting the Alpha variant than the Delta variant (Table 6) and vice versa for the LFIA (Table 7). In support of the findings of this study, Raïch-Regué *et al.* (9) reported a reduced diagnostic performance of Ag-RDTs for the detection of the Alpha and Delta variants compared to the detection of the Omicron variant. Bekliz *et al.* (35) observed a higher sensitivity of the Sure Status COVID-19 Antigen Card Test Kit (Premier Medical Corporation Ltd., India) to the Alpha variant than to the Delta variant, which varies with the findings of this study. However, the Flowflex SARS-CoV-2 Antigen Rapid Test (ACON Laboratories) in the same study showed a higher sensitivity to the Delta variant than to the Alpha variant (35). Contrary to the findings of this assessment, Bekliz *et al*. (36) reported a lower sensitivity of Ag-RDTs for detecting Omicron compared to the earlier variants. The difference in sensitivity of the Ag-RDTs to the different variants can be attributed to the difference in viral load and number of infectious viral particles.

The study emphasizes the proposition of Raïch-Regué *et al*. (9) that the performance of Ag-RDTs for various VOCs depends on the specific antibodies used by each test and viral mutations alone cannot accurately predict their performance. As a result, understanding the viral epitopes recognized by the capture antibodies used by each commercial test is critical to ensuring their efficacy in detecting different VOCs. The sensitivity of Ag-RDTs is exceptionally high when testing is conducted in the first week from symptom onset, resulting in substantially higher sensitivity than testing after 1 week (37). Additionally, Ag-RDTs perform better on samples with lower RT-PCR cycle threshold (Ct) values, indicating a higher viral load (33). These factors contribute to the higher sensitivity of Ag-RDTs in detecting Omicron variant infection, as the variant is known to have a shorter incubation period and higher viral loads than previous variants.

### Conclusion

Antigen detection rapid diagnostic tests are a more affordable and faster alternative to RT-PCR for detecting SARS-CoV-2, making them especially valuable in resource-limited settings. A recent study compared two types of Ag-RDTs, FIA and LFIA and found that the FIA had higher sensitivity, positive predictive value, and accuracy compared to the LFIA. However, the LFIA had a higher specificity and negative predictive value. Both Ag-RDTs showed a strong agreement with RT-PCR, with the FIA performing better in asymptomatic cases and infections associated with the Alpha variant, while the LFIA performed better in infections related to the Delta variant. Notably, both Ag-RDTs demonstrated 100% sensitivity in detecting Omicron infections.

## Data Availability

All relevant data are within the paper and at https://zenodo.org/doi/10.5281/zenodo.10915740.
